# Blood biomarkers for occupational hand-arm vibration exposure

**DOI:** 10.1177/07482337241253996

**Published:** 2024-05-14

**Authors:** Per Vihlborg, Oscar Lundberg, Paul Pettersson-Pablo, Niclas Johansson, Ing-Liss Bryngelsson, Albin Stjernbrandt, Pål Graff

**Affiliations:** 1 Department of Geriatrics, Faculty of Medicine and Health, 596174 Örebro University, Örebro, Sweden; 2 Department of Occupational and Environmental Medicine, Faculty of Medicine and Health, 596174 Örebro University, Örebro, Sweden; 3 Department of Laboratory Medicine, Faculty of Medicine and Health, 59566 Örebro University Hospital, Örebro, Sweden; 4 Department of Ophthalmology, Faculty of Medicine and Health, 596174 Örebro University, Örebro, Sweden; 5 Section of Sustainable Health, Department of Public Health and Clinical Medicine, 59588 Umeå University, Umeå, Sweden; 6 Department of Chemical Work Environment 70672 , National Institute of Occupational Health (STAMI), Oslo, Norway

**Keywords:** Hand-arm vibration, vibration white fingers, Raynauds syndrome, vibration exposure

## Abstract

Hand-arm vibration is a common occupational exposure that causes neurological impairment, myalgia, and vibration-induced Raynaud’s phenomena or vibration white fingers (VWF). The pathological mechanism is largely unknown, though several mechanisms have been proposed, involving both immunological vascular damage and defective neural responses. The aim of this study was to test whether the substances interleukin-33 (IL-33), macrophage-derived chemokine (MDC), interleukin-10 (IL-10), endothelin-1 (ET-1), C-C motif chemokine ligand 20 (CCL20), calcitonin, and thromboxane (TXA_2_) changed before and after occupational hand-arm vibration exposure. 38 full-time shift workers exposed to hand-arm vibration were recruited. All the participants underwent medical examinations regarding symptoms of Raynaud’s phenomena. In 29 of the participants, the concentration of IL-33, MDC, IL-10, ET-1, CCL20, calcitonin, and TXA_2_ was measured before and after a workday. There was a significant increase in ET-1 and calcitonin concentration and a decrease in the CCL20 concentration after the work shift in all participants. In the group suffering from VWF, but not in the non-VWF group, MDC was statistically significantly lower before the work shift (*p* = .023). The VWF group also showed a significant increase in MDC after the work shift. Exposure to occupational hand-arm vibration is associated with changes in ET-1, calcitonin, and MDC concentration in subjects suffering from vibration white fingers, suggesting a role of these biomarkers in the pathophysiology of this condition.

## Background

Hand-arm vibration (HAV) is a common occupational exposure mainly affecting the hands of individuals using vibrating tools (Gemne, 1997). HAV may give rise to Raynaud’s phenomenon (RP), neurological impairment, and myalgia in the hands (Hagberg, 2002; Nilsson et al., 2017). A report from the Swedish Work Environment Study 2021 showed that around 10% of Swedish workers are exposed to HAV at least one quarter of their working time and is a common cause for medical disability (Klevestedt, 2018).

RP is one of the most well-recognized complications of HAV exposure (Bovenzi, 2010; Gemne, 1994). RP caused by HAV is a secondary form of RP, with a known cause that is also referred to as vibration white fingers (VWF) (Prete et al., 2014).

The normal physiological response to a decrease in the external temperature of the hand is vasoconstriction. RP can be described as an abnormal reaction to cold where vasoconstriction impairs blood flow, causing ischemic white fingers (Flavahan, 2015; Prete et al., 2014). RP occurs more often in women, as primary RP (without known cause) or secondary RP in rheumatic disease (Bakst et al., 2008; Belch et al., 2017; Herrick, 2005).

The pathological mechanisms of VWF are largely unknown, with respect to how HAV affects vessels or nerves, causing an abnormal reaction in blood vessels (Nilsson et al., 2017). Several mechanisms have been proposed, involving a maladaptive neural response, increased blood viscosity, endothelial damage from free radical formation, or a direct effect of HAV (Gemne, 1994; Herrick, 2005; Kvietys and Granger, 2012).

Soluble biomarkers related to vasoconstriction and immune system activation have been implicated in secondary RP and can be measured in plasma. Endothelin-1 (ET-1) is a potent vasoconstrictor that is increased in HAV-exposed individuals (Bovenzi et al., 2008; Palmer and Mason, 1996). ET-1 is an antagonist to nitric oxide (NO) (which stimulates vasodilation), and endothelial dysfunction can alter the balance toward vasoconstriction (Marasciulo et al., 2006).

Thromboxane (TXA_2_) is an arachidonic acid metabolite that is involved in platelet aggregation, smooth muscle contraction, and the development of thrombosis (Ally and Horrobin, 1980; Nakahata, 2008; Tarantino et al., 2016). However, TXA_2_ has been found to be elevated in RP secondary to rheumatic disease and is related to the severity of RP (Herrick et al., 1996; Silveri et al., 2001).

Several studies have proposed that activation of the immune system can damage vessels, promoting vasoconstriction (Herrick, 2005; Prete et al., 2014). There is thus reason to believe that chemokines and interleukins associated with the immune system are implicated in the pathophysiology of VWF. C-C motif chemokine ligand 20 (CCL20) is a chemo-attractive substance produced constantly in the body but especially increased in response to inflammation (Schutyser et al., 2003). Interleukin-33 (IL-33) functions as an alarm signal for cellular damage and could be of interest as a marker of tissue injury due to HAV exposure (Cayrol and Girard, 2014). Interleukin-10 (IL-10) is an important cytokine with anti-inflammatory properties that have been found at high serum levels in RP secondary to rheumatic disease (Dziankowska-Bartkowiak et al., 2004; Sabat et al., 2010). Macrophage-derived chemokine (MDC) is involved both in allergic, infectious, and neoplasm pathologies and likely plays a role in other inflammatiory conditions (Mantovani et al., 2000).

Earlier radiographic studies have shown an increase in bone cysts in HAV-exposed subjects, which might be due to microfractures from HAV (Hagberg, 2002). Calcitonin is a hormone that is released to lower calcium in blood during episodes with increased bone resorption and thus could be hypothesized to be involved in VWF pathophysiology (Davey and Findlay, 2013; Felsenfeld and Levine, 2015; Pondel, 2000; Talmage et al., 1980).

## Aim

The aim of this study was to test for early biomarkers that are indicative of a detrimental effect of HAV exposure that might be of diagnostic use in exposed workers. Specifically, this study tested whether the levels of IL-33, MDC, IL-10, ET-1, CCL20, calcitonin, and TXA_2_ changed before and after occupational HAV exposure.

## Methods

### Study population

Thirty-eight (38) full-time shift workers working in a Swedish foundry were recruited. Sixty-eight (68) subjects were invited to participate and 38 accepted (56%). The work tasks that included HAV exposure mainly involved metal grinding. Of the 38 who accepted, six were excluded for lacking hand-arm vibration exposure during the day of blood sampling, with cumulative daily exposure (A (8)) <0.5 m/s^2^, and three were excluded from further analysis of blood samples either because no blood sample was retrieved (*n* = 2) or because of a suspicion of rheumatic disease as the cause for the VWF (*n* = 1).

### Medical examination

The participants underwent a medical investigation using a standardized examination according to Ekenvall, including a medical examination of the neck, shoulders, and elbows and an examination of vascular and neurological function of the hands (Ekenvall et al., 1991). Phalen’s, Tinel’s, and Allen’s tests were performed to evaluate signs of carpal tunnel syndrome and impaired blood flow to the hands. As a complement to the medical examination, all participants answered a questionnaire on symptoms related to hand-arm vibrations the same day before medical examination. This questionnaire is available in Swedish from the occupational medicine method collection (https://fhvmetodik.se/wp-content/uploads/2014/10/frageformular_hand_arm.pdf). VWF cases were defined by being exposed to HAV and having typical symptoms of Raynaud’s phenomenon without any other explanations (e.g., primary Raynaud’s phenomenon or rheumatic disease).

### Sample collection

Whole blood was collected by venipuncture on the right arm, in a vacutainer tube containing an ethylenediaminetetraacetic acid (EDTA) additive, before and after the work shift. Samples were mixed on a rocker for 30 s and then centrifuged at 10°C at 1500 × g for 15  minutes. The resulting supernatant was transferred to an Eppendorf tube and subsequently kept on dry ice at approximately −70°C. At the end of the workday, samples were stored in a −80°C freezer, before being sent to the laboratory in containers containing dry ice. Upon arrival to the laboratory, the samples were immediately transferred to a −80°C freezer where they were kept until analysis.

### Determination of biomarkers

Quantification of macrophage-derived chemokine (MDC or CCL22), CCL20, ET-1, and IL-10 was performed using ELISA kits from R&D Systems (DMD00, DM3A00, DET100, and D1000 B, respectively, Minneapolis, MN, USA). Quantification of TXA_2_, IL-33, and calcitonin was performed using ELISA kits from Biomatik (EKU07649, Kitchener, Ontario, Canada), abcam (ab108918, Cambridge, UK), Thermo Scientific (EHIL33, Waltham, MA, USA), and Cusabio (CSB-E05131h, Houston, TX, USA), respectively. All ELISAs were performed according to the manufacturer’s instructions. The optical densities (O.D.) were read at 450 nm with a wavelength correction at 540 nm in a Multiscan Ascent V1.24 with Ascent Software version 2.6 (Thermo Scientific, Waltham, MA, USA). Data values were expressed as pg/mL deduced from the standard curve after subtracting the blanks, using a 4-parameter logistic algorithm.

### Vibration exposure assessment

To assess the vibration exposure before and after the blood sample was collected, an occupational hygienist performed field measurements at the worksite. The main source of vibration exposure was from grinding machines and exposure was estimated by measuring the vibration level at a point on the handle (or close to where the operator placed his hand) according to ISO 5349-1:2001 (ISO, 2001). A triaxial accelerometer (3023M2 Dytran Instruments, CA, USA) was fastened on a mounting block and the block was attached to the grinder with hose clamps. The handheld four-channel vibration analyzer (Svantek 106, Svantek, Warsaw, Poland) was used to collect data from the accelerometer. The occupational hygienist measured the exposure time during the workday. The vibration level of the tool and exposure duration were then used to calculate A (8) values for all participants.

### Statistical analysis

Descriptive statistics were used to assess the baseline characteristics of the population.

To test blood samples before and after the workday, the Wilcoxon signed-rank test was used to test statistical significance owing to the non-normal distribution of the material. A *p*-value less than 0.05 was considered significant. Statistical software SPSS 25.0 (IBM, North Castle, NY, USA) was used for hypothesis testing.

Written informed consent was collected from all study participants and the Regional Ethical Review Board in Uppsala, Sweden (Dnr 2016/044) approved the study protocol.

## Results

All the participants were male with a mean and median age close to 45 years (mean 44.6, median 45). Employment time was between 1 and 37 years with a mean and median of 13.5 years, respectively (Table 1). The group with VWF had lower mean and median employment time compared to the non-VWF group. Only five participants were smokers but 14 were using snus (non-smoking Swedish tobacco). The tobacco use was lower in the VWF group.

**Table 1.** Descriptive data of the study group, categorized by vibration white finger (VWF) status.

	VWF		non-VWF		Total	
	*N*	%	*N*	%	*N*	%
Sex						
Men	19	100	13	100	32	100
Age						
<=40	6	31.8	5	31.3	11	31.6
41–50	7	40.9	4	31.3	11	36.8
51+	6	27.3	4	37.5	10	31.6
Mean	44.8		45.1		44.6	
Median	45.5		46.0		45	
Min–Max	26–58		28–62		26–62	
Employment year with vibration exposure						
0–5	4	18.2	1	12.5	5	15.8
6–10	5	27.3	2	18.8	7	23.7
11–15	2	9.1	6	37.5	8	21.1
>15	8	45.5	4	31.3	12	39.5
Mean	12.4		15.2		13.5	
Median	12.0		14.0		13.5	
Min–Max	1–33		1–37		1–37	
Smoking						
Never smoking	16	86.5	9	75	25	81.6
Current smoking	2	9.0	3	18.8	5	13.2
Unknown	1	4.5	1	6.2	2	5.3
Non-smoking tobacco^a^						
Never used	13	68.2	6	50.0	19	60.5
Current user	6	31.8	6	44.0	12	36.8
Unknown			1	6.0	1	2.6

^a^Swedish snuff (snus).

The median vibration exposure during the workday measured in 8-h average (A(8)) when the blood samples were taken was 1.8 m/s^2^ for white fingers and 1.3 m/s^2^ for non-white fingers (Table 2). The participants used different tools during the workday, but mainly different grinders, and the exposure time varied between 0.5 and 3 h. The range of HAV exposure for the participants was between 1.0 and 3.1 m/s^2^ A (8).

**Table 2.** Exposure data regarding hand-arm vibration for the study participants during the day of blood sample collection, categorized by vibration white finger (VWF) status.

	VWF	*N*	Mean	Median	Min	Max	Std. deviation
Acute vibration exposure (m/s^2^)^a^	No	12	1.6	1.3	1.0	3.1	0.69
	Yes	17	2.0	1.8	1.0	3.0	0.56
	Total	29	1.8	1.7	1.0	3.1	0.65

^a^Depending on the work tasks during the day measured as an 8-h average (A(8)), the vibration exposure ranged between 1.0 and 3.1 m/s.^2^.

For all 29 participants, there was a significant increase in ET-1 and calcitonin concentration after the work shift. There was also a significant decrease in the CCL20 concentration. For TXA_2_ there was a non-significant decrease and a non-significant increase for MDC and IL-33 (Table 3). From the study group with vibration exposure (*n* = 29) during the day, three was excluded because no blood sample was retrieved or a suspicion of rheumatic disease.

**Table 3.** Changes in concentration of the tested biomarkers during the work shift with hand-arm vibration exposure. The Wilcoxon signed-ranks test was used to calculate the *p*-values before and after work shifts with hand-arm vibration exposure.

		Mean	Median	Min	Max	Std. deviation	*p*-value
Total *N* = 29							
IL-33 (pg/mL)	Before	179.2	7.9	<LOD	682.1	248.3	
	After	180.5	11.7	<LOD	646.4	243.3	0.50
MDC (pg/mL)	Before	539.2	524.1	278.0	803.1	125.7	
	After	555.0	560.6	278.6	782.9	121.0	0.10
IL-10 (pg/mL)	Before	1.7	1.1	<LOD	6.1	1.8	
	After	3.1	1.1	<LOD	25.1	5.1	0.20
Endothelin-1 (pg/mL)	Before	1.0	0.9	0.6	2.1	0.4	
	After	1.3	1.0	0.8	3.7	0.6	**0.01**
CCL20 (pg/mL)	Before	19.3	13.4	4.7	75.0	16.7	
	After	11.8	10.4	3.8	28.3	6.1	**0.02**
Calcitonin (pg/mL)	Before	10.4	10.1	<LOD	34.8	8.5	
	After	15.1	14.4	<LOD	32.8	8.9	**0.04**
Thromboxane A2 (pg/mL)	Before	673.0	333.3	52.7	4019.9	827.6	
	After	650.2	398.2	43.3	4019.9	795.8	0.51

Interleukin-33 (IL-33), macrophage-derived chemokine (MDC), interleukin-10 (IL-10), endothelin-1 (ET-1), C-C motif chemokine ligand 20 (CCL20), calcitonin and thromboxane (TXA_2_), and limit of detection (LOD). LOD for IL-33 0.3 pg/mL, IL-10 0.017 pg/mL, and for calcitonin 0.4 pg/mL. Statistical significant value (*p* < .05) is marked in bold.

There was a significant increase in MDC after the work shift for the VWF group and a non-significant increase for IL-10, ET-1, and TXA_2_ (Table 4). In addition, there was a non-significant decrease in CCL20 for the VWF group. In the participants with no symptoms of VWF (non-VWF), there was a significant decrease in CCL20 that was not observed in the group with VWF. In addition, TXA_2_ was significantly increased after a shift in the VWF group compared to non-white fingers (*p* = .034).

**Table 4.** Changes in concentration of the tested biomarkers during the shift with participants grouped based on vibration white fingers (VWF) status. The Wilcoxon signed-ranks test was used to calculate the *p*-values.

		Mean	Median	Min	Max	Std. deviation	*p*-value
VWF *N* = 17							
IL-33 (pg/mL)	Before	170.9	7.7	<LOD	682.1	264.6	
	After	187.4	11.7	<LOD	646.4	262.9	0.57
MDC (pg/mL)	Before	510.8	509.8	278.0	799.2	121.3	
	After	552.1	550.9	421.7	725.8	87.1	**0.03**
IL-10 (pg/mL)	Before	1.4	1.0	<LOD	4.8	1.5	
	After	1.9	0.9	<LOD	7.6	2.6	0.50
Endothelin-1 (pg/mL)	Before	1.0	0.9	0.7	2.1	0.4	
	After	1.3	1.0	0.8	3.7	0.7	0.08
CCL20 (pg/mL)	Before	20.4	13.4	5.9	75.0	18.8	
	After	12.7	10.1	3.8	28.3	7.4	0.25
Calcitonin (pg/mL)	Before	9.4	11.8	<LOD	16.8	5.8	
	After	14.0	13.9	<LOD	31.8	9.1	**0.03**
Thromboxane A2 (pg/mL)	Before	854.4	618.5	57.7	4019.9	962.4	
	After	880.3	491.5	43.3	4019.9	959.4	0.30
Non-VWF *N* = 12							
IL-33 (pg/mL)	Before	190.9	14.8	1.2	549.0	234.3	
	After	170.7	18.8	1.9	555.1	223.6	0.7
MDC (pg/mL)	Before	579.5	591.5	385.2	803.1	125.6	
	After	559.1	576.9	278.6	782.9	161.8	0.64
IL-10 (pg/mL)	Before	2.0	1.6	<LOD	6.1	2.1	
	After	4.7	1.5	<LOD	25.1	7.1	0.50
Endothelin-1 (pg/mL)	Before	1.0	0.9	0.6	1.9	0.3	
	After	1.3	1.0	0.8	2.6	0.5	0.08
CCL20 (pg/mL)	Before	17.7	13.7	4.7	55.6	13.8	
	After	10.5	10.8	5.2	15.9	3.5	**0.03**
Calcitonin (pg/mL)	Before	11.8	8.8	<LOD	34.8	11.4	
	After	16.7	18.4	1.7	32.8	8.8	0.14
Thromboxane A2 (pg/mL)	Before	415.9	140.3	52.7	1811.9	522.6	
	After	324.2	243.5	44.0	787.6	274.7	0.81

Interleukin-33 (IL-33), macrophage-derived chemokine (MDC), interleukin-10 (IL-10), endothelin-1 (ET-1), C-C motif chemokine ligand 20 (CCL20), calcitonin, and thromboxane (TXA_2_), and limit of detection (LOD). LOD for IL-33 0.3 pg/mL, IL-10 0.017 pg/mL, and for Calcitonin 0.4 pg/mL. Statistical significant value (*p* < .05) is marked in bold.

## Discussion

In this study, we analyzed plasma concentrations in blood samples of workers who were exposed to HAV, and for biomarkers that are believed to be implicated in the pathophysiology of VWF. For several of the analytes, we found changes in the concentration before and after the shift when comparing the VWF and non-VWF groups. ET-1, showing a significant increase for the whole study population, is a known vasoconstrictor, and thus reflects an increased vasoconstriction, possibly due to the exposure from HAV. The baseline concentration of ET-1 did not differ between the VWF and non-VWF groups before the work shift. These findings differed from earlier studies that have shown different results in the baseline, with lower ET-1 concentration in VWF (VWF or other HAV-exposed complications) compared with controls (Eriksson et al., 2020; McKenna et al., 1993). It was theorized that a lower baseline ET-1 can be a compensatory mechanism as a response to vibration damage (Palmer and Mason, 1996). Exposure to cold has similarly been reported to cause an increase in ET-1 (Eriksson et al., 2020; Palmer and Mason, 1996). We found a significant increase for the whole exposed population, which concords with the notion that HAV exposure has a vasoconstrictive effect. Of note is that the daily exposure in this study was relatively low, below the action value of 2.5 m/s^2^, above which the employer is required to take action to reduce the exposure according to EU directive 2002/44/EC.

CCL20 was significantly decreased in the non-VWF group but not for the VWF group when comparing concentrations before and after the shift (Table 3). CCL20 is an inflammatory chemokine that is implicated in several inflammatory conditions (Schutyser et al., 2003). In vitro experiments have found that CCL20 interacts with myostatin. Myostatin enhances the secretion of CCL20, and lower levels might reflect inhibition of myostatin via the myostatin-CCL20-CCR6 axis (Fennen et al., 2021). Myostatin is involved in inflammatory joint diseases and degenerative muscle diseases, and a decrease in Myostatin concentration has the potential to induce an anti-inflammatory response and muscle hypertrophy (Fennen et al., 2021; Gonzalez-Cadavid and Bhasin, 2004). The decrease of CCL20 might reflect an effect of vibration on tissues, inhibiting the inflammatory response to promote muscle tissue repair. This would explain why the results were significant for the whole group but not significant in the VWF group where symptoms of disease were present.

IL-33 and IL-10 showed no changes after exposure, which suggests that these cytokines are not affected by HAV exposure. IL-33 is a signal of cell damage. The lack of change in concentration is consistent with the idea that HAV exposure does not mainly cause cell damage, and that the main pathophysiological mechanism is related to vasoconstriction (Cayrol and Girard, 2014). IL-10 increased non-significantly, and other studies have found a higher serum level to correlate with RP symptoms in systemic sclerosis patients with newly debuted RP, suggesting its involvement in the early stages of RP (Dziankowska-Bartkowiak et al., 2004).

There was a significant increase in calcitonin for the whole study group and VWF group after the work shift and a non-significant increase in the non-VWF groups. Calcitonin decreases serum Ca^2+^ by bone resorption via osteoclasts and enhances Ca^2+^ excretion by the kidneys (Pondel, 2000). HAV exposure is suspected to induce bone cysts via microfractures, especially from single impacts (when working with impact tools), even if the results are conflicting (Hagberg, 2002). An increase in calcitonin could be a response to an increased blood Ca^2+^ from microfractures from HAV exposure (Paavolainen et al., 1989; Xie et al., 2020).

As for MDC, there was a significant increase in the VWF group after exposure and a non-significant decrease in the non-VWF group. There was also a lower concentration in the VWF group compared to the non-VWF group. MDC is produced by monocyte-derived dendritic cells and has chemotactic effects to attract immune cells (Godiska et al., 1997). Macrophages and other innate immune system cells have been suggested to play a pathogenic role in early scleroderma—a disease that has similar symptoms to VWF (Godiska et al., 1997). Our results could be explained by MDC attracting innate immune cells that have pathogenic effects (Chia and Lu, 2015). Reports of serum levels of MDC being increased in systemic sclerosis, but not in systemic lupus erythematosus (SLE)—both diseases that include symptoms of RP—could be indicative of differing mechanisms in the pathogenesis of RP, depending on the disease (Fujii et al., 2004). This suggests that systemic sclerosis and VWF share pathophysiologic features that differ from those of SLE.

A non-significant increase in TXA_2_ was observed for all study groups. However, both before and after exposure, higher concentrations were observed in the VWF group compared to the non-VWF group. This contradicts earlier studies showing no difference in TXA_2_ concentration between VWF and controls (Eriksson et al., 2020; Nowak et al., 1996). In primary RP patients, increased TXA_2_ production from platelets induces vasoconstriction (Polidoro et al., 2012). Our results could suggest that participants with VWF can have a tendency for primary RP or more active platelets that produce TXA_2_. In other studies, TXA_2_ has been found elevated and increased with the severity of the RP that is secondary to rheumatic disease (Herrick et al., 1996; Silveri et al., 2001). The increase in TXA_2_ in our result might be due to those exposed to HAV acquiring an ischemic environment because of vasospasm in the fingers which, via ROS production, increases TXA_2_—which could then explain why there was no increase in concentration during the shift for the VWF group where there already was an ischemic environment (Lau et al., 1992).

A strength of this study is that all participants were medically examined by the same physician and under the same circumstances. Samples were collected before and after shifts regardless of the time of day (morning, afternoon shifts, and night shifts). The HAV exposure was measured in all participants during the day, between the collections of the blood samples. The limitation of this study is the rather small study population, which was stratified into two groups. We also did not have data on HAV exposure during the days prior or non-work-related exposure in the study, which could affect the baseline values of the biomarkers. The daily exposure from HAV in our population was below the action value set by government regulation, of 2.5 m/s^2^ A (8), and the low levels of exposure could be too low to detect changes in physiology or plasma biomarker concentrations. On the other hand, our results call the accepted limit of exposure into question, as levels of exposure below the cut-off (median exposure of 1.8 m/s^2^) had measurable, statistically significant effects on the exposed group. This is in line with other studies that reported adverse health effects below the current action limit of 2.5 m/s^2^ A (8) (Clemm et al., 2020; Nilsson et al., 2017). We also did not have a control group for confounding factors such as ergonomics, airborne particles, noise, or skin contamination from industrial exposure. This type of foundry had no silica exposure and air pollution was mainly iron dust from grinding. Owing to the small sample size, the results could not be stratified for age, or tobacco use. However, all participants were instructed to abstain from tobacco products one day prior to the examination. Our results warrant further studies, in which health outcomes in individuals subjected to exposures below the legislative limit are studied, in order to assess the risks associated with lower exposures and whether the limit should be adjusted.

## Conclusion

HAV exposure is associated with significant changes in ET-1 and calcitonin concentration, which suggests that vasoconstriction and Ca^2+^ metabolism are involved in the response to HAV, even at low levels. In the VWF group, an increase in MCD compared with the non-VWF group suggests that MCD is involved in VWF pathophysiology. Despite exposures below the action value, physiological and biomarker changes were observed, which calls the suitability of the current limit level into question. This study showed that some of the biomarkers have the potential to be useful to both study early effects of vibration exposure as well as for medical surveillance of exposed workers in the future.

## Data Availability

The data used in this study were derived from patients. Any researcher, granted that they have an ethical approval from a regional ethical board, can contact the Department of Environmental and Occupational Medicine at Örebro University Hospital (USÖ) for the study data. However, the Swedish National Board of Health and Welfare will also put restrictions on sharing sensitive information (www.socialstyrelsen.se/en/).
